# When Health Systems Are Barriers to Health Care: Challenges Faced by Uninsured Mexican Kidney Patients

**DOI:** 10.1371/journal.pone.0054380

**Published:** 2013-01-22

**Authors:** Ciara Kierans, Cesar Padilla-Altamira, Guillermo Garcia-Garcia, Margarita Ibarra-Hernandez, Francisco J. Mercado

**Affiliations:** 1 Department of Public Health and Policy, The University of Liverpool, Liverpool, United Kingdom; 2 Division of Nephrology, Hospital Civil de Guadalajara, University of Guadalajara Health Sciences Centre, Hospital 278, Jalisco, Mexico; 3 Depto Salud Pública, CUCS, Universidad Guadalajara, Guadalajara, Jalisco, Mexico; University of Sao Paulo Medical School, Brazil

## Abstract

**Background:**

Chronic Kidney Disease disproportionately affects the poor in Low and Middle Income Countries (LMICs). Mexico exemplifies the difficulties faced in supporting Renal Replacement Therapy (RRT) and providing equitable patient care, despite recent attempts at health reform. The objective of this study is to document the challenges faced by uninsured, poor Mexican families when attempting to access RRT.

**Methods:**

The article takes an ethnographic approach, using interviewing and observation to generate detailed accounts of the problems that accompany attempts to secure care. The study, based in the state of Jalisco, comprised interviews with patients, their caregivers, health and social care professionals, among others. Observations were carried out in both clinical and social settings.

**Results:**

In the absence of organised health information and stable pathways to renal care, patients and their families work extraordinarily hard and at great expense to secure care in a mixed public-private healthcare system. As part of this work, they must navigate challenging health and social care environments, negotiate treatments and costs, resource and finance healthcare and manage a wide range of formal and informal health information.

**Conclusions:**

Examining commonalities across pathways to adequate healthcare reveals major failings in the Mexican system. These systemic problems serve to reproduce and deepen health inequalities. A system, in which the costs of renal care are disproportionately borne by those who can least afford them, faces major difficulties around the sustainability and resourcing of RRTs. Attempts to increase access to renal therapies, therefore, need to take into account the complex social and economic demands this places on those who need access most. This paper further shows that ethnographic studies of the concrete ways in which healthcare is accessed in practice provide important insights into the plight of CKD patients and so constitute an important source of evidence in that effort.

## Introduction

Chronic Kidney Disease (CKD) is a global public health concern. CKD is the 12^th^ leading cause of death worldwide [1], its incidence growing by approximately 8% annually [2]. Low and middle-income countries are facing increasing difficulties supporting resource-intensive Renal Replacement Therapy (RRT) and providing equitable patient care [3], [4]. In countries characterised by extreme health inequalities, unregulated medical environments [1] and an absence of comprehensive kidney registries, the true burden of CKD remains difficult to assess [5]. Furthermore, CKD has a more complex aetiology than in industrialised countries. In addition to the established risk factors of diabetes and hypertension, infectious and environmental causes create challenges for prevention monitoring and treatment [6], [7], [1]. The scale of the problem and the difficulties associated with its management have generated intense scientific debate about how to deliver cost-effective treatment through targeted health reform in under-resourced contexts [8], [1]. Mexico, the focus of this article, is one country which exemplifies the challenges faced by low and middle-income countries in managing CKD.

### CKD in Mexico

The prevalence of CKD in Mexico is similar to industrialised nations, while the resources for its treatments lag significantly behind [9], [7]. With close to 9% of the population diagnosed with CKD [9], [11], [12], and 60,000 patients on RRT [13], [10], the condition is rapidly becoming a major national concern, particularly with regard to inequalities of access to treatment [12], [10].

Health inequalities in Mexico are commonly linked to its fragmented, state-corporatist system of health care provision. This system generates problematic distinctions between insured and uninsured populations. Workers in the formal economy, approximately 55–60% of the population, are covered by an insurance infrastructure administered by social security institutes. IMSS (Instituto Mexicano del Seguro Social (Mexican Institute of Social Security)), is the largest, accounting for 82% of those with health insurance [14]. The remainder of the Mexican population has no coverage and relies on subsidised services and programmes provided by clinics, hospitals and various programmes run by the Health Secretariat. These services are run at a significant out-of-pocket cost to the poor who use them [15], [16]. Reforms designed to alleviate the catastrophic poverty produced by an inadequate public health infrastructure have been pursued, most recently through the development of *Seguro Popular* (Popular Health Insurance). Advanced in 2001 as an integrated health insurance programme for the poor, Seguro Popular was to be funded by federal and state subsidies and a means-tested premium paid by families, who were to enrol on the programme on a voluntary basis. It aimed to develop an explicit package of services, facilitate greater market competition and shift the federal budget to demand-based allocation of services [17], [18]. The underlying assumption was that access to medical care would be enhanced through market discipline and a greater distribution of costs [19]–[23]. Seguro Popular's development, implementation and evaluation received extensive coverage by the Lancet in 2006, and again most recently in July 2012, where it has been presented as *the* route to universal health coverage and social protection, an evidenced example, one that other low and middle-income countries can learn from [24]–[29]. While Seguro Popular has increased access to care for some conditions (though not for CKD, End Stage Renal Disease or their treatments), its critics argue it has not reduced inequalities in access, but is serving to further fragment and stratify Mexico's health system, exacerbating inefficiencies in delivery [17], [20], [21], [30], [31], [32].

One of the difficulties researchers and analysts currently face in assessing the merits and limits of reforms such as Seguro Popular, is the absence of evidence around their translation into practice. Existing evaluations have tended towards broad health systems analysis, provided in the main by quantitative overviews, and often conducted by those also charged with implementing reform [33], [34]. They tell us little about the challenges faced by patients and their families in trying to access health care, or indeed by health professionals in attempting to ensure equity and quality of service provision. In order to develop equitable access strategically, it is imperative that researchers fully account for the situated experiences of health care in addition to synoptic overviews, when attempting to understand how health systems perform in practice [35].

By drawing on the contributions of ethnographic approaches, we aim to provide an account of this kind. Ethnographic research is a form of “social research based on the close-up, on-the-ground observation of people and institutions in real time and space in which the investigator embeds herself near (or within) the phenomenon, so as to detect how and why agents on the scene think and act the way they do” [36].

The paper, therefore, has two aims: (1) to put forward an ethnographic framework for analysing patterns of healthcare access at local levels and (2) to demonstrate the challenges and barriers encountered by uninsured CKD patients when attempting to access health services.

## Methods

### Setting

The setting for the research was Hospital Civil de Guadalajara in the State of Jalisco, Mexico. It is a large tertiary facility which has served Jalisco's poor since 1792. The hospital operates from state and federal funds, private donations from individuals and contributions from non-governmental organizations. However, it functions as an independent health care provider, with its own budget and board of directors. The hospital is unique in Mexico. It is the country's largest dialysis and kidney transplant centre for those without health insurance, and it is the only facility which subsidises both haemo- and peritoneal dialysis for the poor. Outside of that, patients are charged according to their level of income. The hospital has been home to a transplantation programme since 1990. It runs a large peritoneal dialysis programme – the main modality of renal care in Mexico [37] – and a haemodialysis unit which, due to limited resources, is used as a back-up to the peritoneal programme [38].

The decision to locate the study here was due to the hospital's stated commitment to patients without health insurance, but also because the city, more generally, is home to a number of successful renal care and transplantation programmes. Nephrologists based here established the Jalisco State Dialysis and Transplantation Registry in 1993 the only one of its kind in Mexico [9]. This has helped to keep CKD, particularly End State Renal Disease under epidemiologic surveillance and generate data that index CKD and its treatment nationally and internationally [39]. All public, social security institutions participate, such as IMSS, ISSSTE (Instituto de Seguridad y Servicios Sociales de los Trabajadores del Estado (Institute for Social Security and Services for State Workers)), the Hospital Civil, Guadalajara and institutions belonging to the Health Secretariat. This generates data on approximately 90% of the dialysis and transplant state population. All patients who started on renal replacement therapy between January 1998 and December 2010 at these social security facilities are included. At the start of dialysis, patients' social security or insurance institution is registered along with age, gender, aetiology of renal disease and treatment modality. Unadjusted incidence and prevalence rates are reported and data on the number of nephrologists and dialysis and transplant facilities are annually updated (information on the methods used by the registry are in the public domain [40] and the quantitative data collected by the registry has provided an important statistical backdrop to the ethnographic, qualitative methods used in this study).

Inequalities of outcome between CKD patients with and without insurance in Jalisco are, as a result, well-evidenced and have provided impetus for a series of strategic interventions aimed at reducing inequity and supporting improvements in screening and prevention [40], [12]. In Jalisco, those without insurance suffer high mortality when end-stage renal disease develops, with approximately half dying within 6 months of dialysis therapy initiation [37]. For insured patients, acceptance and prevalence rates are considerably higher (478 per million population (pmp) and 1211 pmp, respectively) than the uninsured (231 pmp and 286 pmp); for rate of kidney transplantation, this is 82 pmp versus 20 pmp; for provision of dialysis units 21 versus 3; and for number of nephrologists 12.1 pmp versus 3.4 pmp [12]. Social Security covers 44.2% of the population in the state and a further 3.5% are covered by civil service and private schemes. While 15.6% are identified as having *Seguro Popular*, 36.6% are left without any coverage or benefits [41]. These latter figures, however, only relate to those who have chosen to enrol in Seguro Popular. As Seguro Popular provides no coverage for renal replacement therapies, not all patients will register.

### Ethnographic Approach

Data for this paper was collected between June and September 2011 and again during July and August 2012 as part of a collaborative ethnographic study carried out by researchers at the University of Guadalajara and the University of Liverpool, UK with support from clinicians at the Hospital Civil de Guadalajara. The advantage of approaching the problem of inequalities ethnographically was threefold: (1) Ethnographic research, is dependent (though not exclusively) on in-depth qualitative interviewing and forms of observation. This facilitates a clear focus on what people do, how people understand what they do and how such practices are embedded in particular cultural, social, economic and political contexts. (2) It pays attention to the connections between different areas of social life (like illness, the family and work) rather than separating them off as domains to be studied discretely. In the case of CKD, it concentrates on the contexts within which patients and their families attempt to access health care and traces the work families do vis-a-vis other related health and social care agents, such as health care professionals, policy makers, social security administrators, charitable and voluntary associations. (3) Ethnography, finally, takes into consideration socio-economic and political structures which play an integral role in healthcare. In this case, adopting an ethnographic approach made it possible to follow the problem of CKD to show how health care is navigated from below as well as administered from above. It thus describes how these systems operated in practice [42].

### Data Collection

The study comprised 105 interviews incorporating 138 respondents, 32 of which were in-depth narrative style interviews and 73 were semi-structured. They included 51 renal patients across all modalities of treatment, 34 caregivers, donors and family members and 53 interviews distributed between health and social care professionals, patient support groups/charitable organisations and medical suppliers as well as Mexican scholars writing on this subject area (*please see* table 1). The respondents were purposely selected to obtain a maximum variation sample. In addition to interviews, observational research was carried out in the hospital: at medical consultations, in hospital wards and in related settings, such as patients' homes and patient support meetings and events. Approximately 200 pages of field notes were written, providing contextual information to ground the analysis of the qualitative interviews, as well as documenting the fieldwork process. The research strictly adhered to the ethical codes of practice set out in the requirements of the American Anthropological Association and the Association of Social Anthropologists, UK, which reflect the particularities of conducting ethnographic fieldwork. Both written and verbal consent were taken. Consent was verbal in cases where participants were uncomfortable providing a signature on forms, suspicious of bureaucratic processes or during informal ethnographic interviewing where it was not always appropriate to request written consent. In every case, we took time to explain the objectives, procedures and possible outcomes of our research. We asked permission to record the interviews, gave clear assurances on anonymity, confidentiality and the right to withdraw at any stage. Participants' queries and questions were answered before and after interviews. Both written and verbal consent procedures complemented each other in the process of ensuring an ethically robust study and were documented through the writing of field notes, a process, in this case, overseen collectively by the researchers involved. Ethics approvals for ethnographic fieldwork, which incorporates both formal and informal interviewing and written and verbal consent recorded in this way, were awarded by the University of Liverpool and the Hospital Civil de Guadalajara research ethics committees.

**Table 1:** Study Participants.

Participants	Total
Patients	51
Caregivers, donors and family members	34
Physicians	16
Social workers	7
Nurses	7
Psychologists	1
Nutritionists	1
Organ donor coordinators	1
Patient support associations	7
Health decision makers	4
Pharmaceutical and laboratory representatives	5
Pharmacists	1
Academic scholars	3
Total participants	138

### Analysis

This mix of interviewing and observation, which was carried out across different settings and across all our participants - patients, caregivers, health professionals and so on - yielded highly detailed and complex accounts of the process of accessing renal care. While these accounts are characterised by a diversity of patient and family experiences and histories, we were particularly interested in identifying ‘structural’ commonalities among the types of processes found across all cases: including the ‘built-in’ constraints on the kinds of pathways patients had to follow through the system and the kinds of demands placed on them as well as the resources they were required to expend as they did so. As a result, and for the purpose of this particular paper, we focused our attention on these commonalities, concentrating on the trajectories or journeys of healthcare seeking for CKD patients in Mexico. This has enabled us to effectively demonstrate the profound challenges poor families face, and the strategies they employ, in accessing renal care and managing CKD [43]. As part of reading through and analysing all our data, we identified the recurrence of various kinds of steps and actions taken by patients and their families, such as the forms of exchange underpinning access to health care, interactions with various personnel within pertinent clinical sites, as well as the wide range of resources that patients draw on to secure care. The three cases that were finally chosen were selected for their typicality, in that they usefully exemplified the kinds of structural problem which emerged across our data as a whole. The value in taking an ethnographic approach to the problem of accessing care is not to abstract away from the richness of real world cases but to show, with reference to them, what can be drawn out in order to help us make sense of further cases. More conventional approaches which rely on the use of interview quotations would not help us to achieve this. In fact, it would carve up the process of seeking care much more idiosyncratically, making it difficult to extrapolate beyond individuals' perspectives or subjective experiences. It is also very important to add that while we do not specifically draw out individual points-of-view from the rest of our informants, such as health professionals or members of charitable organisations, our analysis has been informed and developed in relation to their input: our many discussions with them and observations of the work they do. Their input has served to strengthen our analysis, as we have not simply relied on one constituency of informant. Developing a robust account of processes which are complex, messy and discontinuous in practice has, thus, relied on an analysis of the structural features of each ‘case’. Our analysis was also informed by the rich legacy of ‘interactional approaches’ [44] to studies of health care and owes much to the well established concept of the patient ‘career’ [45] which has helped us develop our understanding of the significance of recurrent or ‘typical’ characteristics within the experience of healthcare.

## Results

The cases relate to three transplant recipients (Elena, Emilio and Gabriel), all aged between 18–22. They provide insight into how families access care within the distributed and complex Mexican system, across public and private institutions, while attempting to find the appropriate financial, human and moral resources to do so. All were interviewed with family members, in the hospital and in their homes. All were from large working class families, with average annual incomes well below the average in Jalisco of approximately 81,430 pesos (approx 6,000 US dollars). None had a medical explanation as to why they had CKD. Emilio and Gabriel received their kidneys from their mothers, Elena from her older sister. Elena and Emilio had been on haemodialysis and Gabriel was on peritoneal dialysis. All had attempted to use Seguro Popular for different aspects of their health care, but learned in the course of seeking support, that it did not cover CKD or its treatments. All had come to Hospital Civil because of its reputation for quality care for poor patients. Descriptions of these cases can be found in tables 2, 3, 4.

**Table 2:** Elena, her mother and sister Rita.

Elena was an 18 year old transplant recipient. She received a kidney from her older sister Rita in March 2011. She had been diagnosed with CKD in Hospital Civil in 2009, after attending three private physicians and another public hospital. Unsuitable for peritoneal dialysis, she was put on haemodialysis. Because her parents had IMSS insurance, the family were initially sent to IMSS facilities, only to find that Elena, no longer formally at school, was not covered.On medical advice, she went to a private hospital, known for providing low-cost dialysis ($84 USD/session). To help finance, the family were given a letter by a doctor to go to DIF Jalisco (Desarrollo Integral de La Familia – a national public assistance organisation with Federal, State and Municipal offices). Support was provided for four free sessions, after which the family went to Caritas Mexico, (the international Catholic social welfare provider), followed by the DIF Zapopan municipal office, then by DIF in Guadalajara. Each provided payment for a few dialysis sessions. Elena's mother took on the role of sourcing financial support, while her sister negotiated her treatments and care. After three months of dialysis, they moved to another private clinic run in conjunction with a pharmaceutical company, with support from DIF Guadalajara and a philanthropic organisation.One year into dialysis, the family made an ‘informal’ arrangement with a cleaning company to ‘hire’ Elena, so that she could qualify for IMSS insurance. Taking advantage of such slippages in an overburdened insurance system was not uncommon among patients. The family, in turn, agreed to pay the employer and employee's insurance contribution, however this lasted only as long as they could afford the premiums. With this in place, Elena received the remainder of her dialysis (two more months) free of charge at an IMSS affiliated hospital.Attempts to secure a transplant were particularly difficult. IMSS could not transplant her due to long waiting times and after much negotiation, Hospital Civil agreed. The family would have to pay for all required pre-transplant tests, many out-sourced to private laboratories (approx $1,400 USD). To meet the costs, the family sold their land; appealed to everyone they knew; requested money from relatives in the US and petitioned local TV stations and businesses.Elena was transplanted at a cost of approximately $1,241 USD (surgery only). The family, now penniless, went to a money lender to borrow money for immunosuppressants. They were finding it increasingly difficult to find the $37 USD for post-transplant monitoring and checkups, not to mention the $15 USD extra charges for taxi fares. Elena had complications post-transplant and had to be rushed back into hospital. With her family stripped of all resources, her sister said: “We are now between the sword and the wall, we don't have money for Elena's post-transplantation care or to pay the money lender. We don't know what we are going to do”.

**Table 3:** Emilio and his mother Ana.

Emilio was 20 years old and unemployed. He was diagnosed with CKD at 14, his condition managed at a public hospital near his home, until kidney functioning reached ‘end stage’. He then moved to Hospital Civil. He spent ten months on haemodialysis there, paid for by money raised by his family, while the necessary protocols prior to transplantation were conducted.Emilio received a kidney donated by his mother, three months prior to interview. Since then he had been sick. His doctors weren't sure whether he was experiencing toxicity, rejection or a virus, and requested a biopsy. His mother was asked to purchase a biopsy needle ($146 USD), while Emilio was hospitalised, waiting for the biopsy. After this his mother was expected to bring the biopsied sample and Emilio's medical files for private clinical analysis ($365 USD). The biopsy had to be re-scheduled. The family were charged $132 for hospitalisation, Emilio's mother complained, and they secured a renegotiated price of $44.Without insurance, the family relied financially on relatives working as cleaners and labourers in the US; on more immediate family to provide blood donations and smaller amounts of money; on a range of charitable donations and on fellow patients for advice for obtaining discounts on medications and sourcing less expensive laboratories for analysis. Since his transplant, Emilio's family have made an ‘arrangement’ with a neighbour who owns a corner shop to formally employ Emilio so that he qualifies for IMSS insurance which covers the cost of immunosuppressants.Ana, his mother explained that resources are difficult to find morally and economically. Aside from being Emilo's donor and carer, she also looks after her husband, a labourer, who has prostate cancer. She says, “There are no rights for kidney patients … it is a tragedy because lots of young people are getting sick with this condition …. Seguro Popular doesn't cover us. They send you to the gutter. They don't want to know – they will give you some drugs but not the expensive ones.”

**Table 4:** Gabriel and his mother Maria.

Gabriel is a college-level teacher from Chapala (62 kilometers from Guadalajara). He was diagnosed with kidney disease in 2008 and put on peritoneal dialysis (PD). For this, his family constructed an extra room from plywood within the existing space of their living room, at great personal cost. This had to be specially painted, kept immaculately clean and equipped with microwave, weighing scales and countless boxes of dialysate solution.Gabriel's teaching job should guarantee IMSS insurance but he was told he would not be covered as his CKD was pre-existing. This, however, was not correctly communicated, but instead reflected a failure within his school to understand and explain adequately his level of social protection. To assist with the ensuing costs, support was provided by a relative who ran a local clinical laboratory, and gave them credit on medical tests. Further help was given by families, who had lost a member due to CKD, by establishing an informal distribution network of unused solution, disinfectant and medications.After three years on PD, in June 2011, Gabriel received a kidney from his mother, but rejected the graft only a few weeks prior to our interview. He was back on PD and awaiting a cadaveric organ. Rejection was explained as a result of a miscommunication regarding the amount and type of immunosuppressants he would need. The family had, previously, raised the money for the transplant from fundraisers and family in the US, but were not prepared for the cost of immunosuppression. His mother, who cried when she talked of the kidney rejecting, explained that travelling to the hospital leaves no money to buy food as everything went to pay for routine check-ups, consultations and the bus fare. A few weeks prior, they were offered the chance of a cadaveric kidney. His mother explained:“They (doctors) said come today, you can pay tomorrow, but I explained it isn't that easy. He (doctor) said you need only $365 USD for the cross-match tests. I said, but we know how much everything really costs, so we said no, we will wait until we have the money. He said, are you going to waste the opportunity, and we said, yes. A transplant … for everything … is somewhere between $3,600–4,380 USD. Everything has to be bought, even the material for stitching the wound. It is too much for a family. … And Seguro Popular, we don't have it. For us, it only covers consultations and one or two pills. Even the doctors complain about it. It doesn't make sense, there is no point in it. It's just a government lie”.

## Discussion

Exemplified by these cases and the wider interviews, four key practices within the trajectories of health care seeking have been identified as underpinning the ‘work’ families in Mexico must do to secure health care. These are described in table 5.

**Table 5:** The Practical Work done by Mexican CKD Patients and their Families.

Practices	Description
**Navigating health and social care structures**	Without any synoptic overview of health care provision or any established patient pathway, families must identify appropriate healthcare institutions in order to acquire a diagnosis, form of dialysis and secure a transplant. This routinely involves an unscripted set of journeys or zigzagging between various public and private health care providers, clinics and laboratories.
**Negotiating treatments and costs**	Without health insurance, families must pay for all aspects of their medical care – hospitalisation, surgeries, consultation, routine check-ups and tests, dialysis, pre-transplantation protocols, biopsy needles, stitching for wounds, disinfectant, antibodies, surgical procedures, among a wide range of medications e.g., erythropoietin and immunosuppressants. In addition, there are travel costs, dietary costs, structural housing costs for peritoneal dialysis patients, informal care giving and the loss of formal earnings. Some of these costs are negotiable as a result of lobbying social workers and medical staff, or partially covered by ad hoc insurance coverage.
**Financing and resourcing health care**	Financing CKD and its treatments requires ingenuity and hard work and involves appeals to family and neighbours, fund raising, negotiating with charitable and voluntary associations, selling land and inheritances, begging, appealing to local business or the media.
**Managing formal and informal health information**	Due to a lack of integrated administrative systems within and between hospitals, patients carry their own medical files and test-results to all appointments. This produces a burden of information that has to be understood, processed and disseminated regularly. Furthermore, it serves to shift the burden of responsibility onto the patient, making them the principal agent in the management of their health care, rather than the state or any ‘sited’ health care provider.

Taken together these practices constitute responses to the disintegrated character of care for the uninsured CKD patient in Mexico, drawing attention to its lack of structure and clear pathways to treatment. From the point of diagnosis, there is little information and no clearly defined administrative infrastructure. This means that families have to find their own routes through various public and private health care providers, laboratories, pharmacies, social support organisations and so on. In effect, each family has to ‘make’ a health care system for themselves, connecting together treatment as they go. That there is no financial coverage for this condition, despite the rhetoric of Seguro Popular's increasing coverage for the poor, means that every aspect of renal care has to be resourced by the family. Not only does this serve to make poor families poorer, it entrenches social and health inequalities, particularly gender inequalities, as the burden of care is disproportionately taken on by women as organ donors and carers as well as financial negotiators for health care.

What is evident in the work families do to negotiate access to health care is that the rhetorical distinctions between insured and uninsured break down and conflict in practice. This is demonstrated by the ways in which patients move between public and private providers and their associated insurance systems, depending on need, waiting times, quality of care and cost. For patients relying on renal replacement therapy, the consequences of this ‘zigzagging’ are inconsistency of care, difficulties in maintaining drug regimens, and making routine check-ups and monitoring. This profoundly threatens the sustainability of transplanted organs, due to infections, graft rejection and the haphazard taking of immunosuppressants.

Given efforts to increase access to renal replacement therapy for Mexico's poor at Hospital Civil, integrated care is of vital importance. In 2011, Hospital Civil had approximately 240 patients on peritoneal dialysis, 70 patients on haemodialysis and performed 53 transplants. This reflected an increase in capacity by the hospital, which had in May of 2011 opened a new, modern haemodialysis facility. Despite its limited resources, Hospital Civil remains one of the few centres in Mexico providing comprehensive renal care to the uninsured. However, in a context where families have become responsible for their own health care, ensuring access to renal therapy is not enough, particularly when sustainability and continuity of treatment cannot be guaranteed. This raises critical questions about the importance of resourcing, not only in Mexico, but across low and middle-income countries. In particular, it raises questions about the manner in which costs are currently distributed between state, industry and society, and how these costs could be better borne by these parties to ensure greater equity and justice in health care.

A potential limitation of this study may reflect the fact that the experiences of our participants may not be representative of the specific experiences of individuals who seek renal care at other public institutions in other states. Nevertheless, due to the ways in which treatment is configured in Mexico, we can point to wider lessons to be learned. Our approach has attempted to provide an account of the structure of interactions with the public health system and to focus on commonalities across the variable trajectories of individual patients. Given that Seguro Popular is supported by federal government and that this form of health care delivery is reflective of the Mexican system as a whole, it is reasonable, therefore, to suggest that the structuring of access to health care and the types of practices we have documented will be found in other states, and potentially across other conditions.

## Conclusion

CKD disproportionately affects the poor and the socially disadvantaged, particularly in low and middle-income countries. Its burden is not limited to access to renal replacement therapy but to overall population health [46]. It is known to exacerbate other chronic conditions and impacts profoundly on mental health, working capacity and family life. All predictions show that CKD and its impacts are set to worsen over the coming decades [2]. In the interest of enhancing equity in health care, this has given rise to discussions about the importance of prevention [2], the necessity of low cost drugs [1] and the different roles to be played by the state, private sector, charitable organisations and citizens in the work of healthcare [46], [47]. However, in order to gauge the effects on the most vulnerable, we cannot rely solely on synoptic views of health systems, medical interventions and outcomes to draw conclusions about equity or, indeed, as the basis for evaluations. This is of particular concern in low and middle-income countries engaging in health reform, as processes which may be planned to simplify and rationalise healthcare provision may actually massively complicate the processes of accessing care for those who need it most. On paper, current developments in Mexico are opening up CKD treatment to the poor. In practice, these developments are caught within a system which is leading to further impoverishment, as patients are caught in a ‘medical poverty trap’ [48]. In order to respond to the problems the poorest face in practice, we must attend to how systems of healthcare work in practice. This means tracing the impacts of healthcare reform, medical interventions and patient outcomes in local contexts.
