# Correction: When Health Systems Are Barriers to Health Care: Challenges Faced by Uninsured Mexican Kidney Patients

**DOI:** 10.1371/annotation/453f8721-a450-469c-9fd7-5cf43b21f957

**Published:** 2013-10-25

**Authors:** Ciara Kierans, Cesar Padilla-Altamira, Guillermo Garcia-Garcia, Margarita Ibarra-Hernandez, Francisco J. Mercado

The Funding statement in the article was incorrect. The correct version of the Funding statement is available below.

The authors acknowledge the following sources of funding: The University of Liverpool, UK provided research costs; The University of Guadalajara, Mexico provided research costs and the Peter Lienhardt Memorial Fund and Philip Bagby Bequest from the University of Oxford, UK provided a travel grant. The funders played no role in study design, data collection and analysis, decision to publish, or preparation of the manuscript. 

